# Osteomalacia and renal failure due to Fanconi syndrome caused by long-term low-dose Adefovir Dipivoxil: a case report

**DOI:** 10.1186/s40360-020-00421-6

**Published:** 2020-06-05

**Authors:** Qian Xiang, Zhiyan Liu, Yanyan Yu, Hanxu Zhang, Qiufen Xie, Guangyan Mu, Jianhua Zhang, Xinan Cen, Yimin Cui

**Affiliations:** 1 Department of Pharmacy, Peking University First Hospital, No. 6, Dahongluochang Street, Xicheng District, Beijing, 100034 China; 2 School of Pharmaceutical Sciences, Peking University Health Science Center, Beijing, China; 3 Department of Infectious Disease, Peking University First Hospital, Beijing, China; 4 Nuclear Medicine Department, Peking University First Hospital, Beijing, China; 5 Department of Hematology, Peking University First Hospital, No. 6, Dahongluochang Street, Xicheng District, Beijing, 100034 China

**Keywords:** Adefovir dipivoxil, Fanconi’s syndrome, Osteomalacia, Case report

## Abstract

**Background:**

Progressive bone pain and fracture and abnormal positron emission tomography combined with a computed tomography are main reasons for the oncologists suspecting bone tumor. During the patient’s medical treatment, the oncologists’ unfamiliarity with adverse reactions to anti-HBV drugs were main reason for the long-term exposure to the drug and the adverse reaction (ADR) experienced by the patient.

**Case presentation:**

A 63-year-old Chinese man had a 27-month history of progressive generalized bone pain combined with spontaneous fractures. Positron emission tomography combined with a computed tomography, revealed an abnormal increase in ribose metabolism and low positron serum inorganic phosphorus concentration (0.7; 0.78–1.65 mmol/L). Serum creatinine level was 252 μmol/L (53–97) μmol/L, and glomerular filtration rate was 22.79 mL/min/1.73 m^2^. The patient was referred to a multidisciplinary clinic to clarify the diagnosis of myeloma or bone tumor for further treatment in 2017. His medical history revealed that he had a 30-year history of chronic hepatitis B infection. He had received lamivudine at a daily dose of 100 mg for 19 years (1990 to 2009), which had been changed to adefovir (10 mg/day) owing to lamivudine resistance in 2009. Based on the changes in the patient’s laboratory markers and the results of emission computed tomography and other radiographic findings, adefovir-induced hypophosphatemic osteomalacia due to acquired renal Fanconi syndrome was suspected by the clinical pharmacist. Considerable clinical improvement was observed after adefovir discontinuation and the administration of entecavir (1.0 mg, every other day).

**Conclusion:**

Fanconi syndrome with osteomalacia can develop in patients with chronic hepatitis B infection being treated with adefovir at a conventional low dosage of 10 mg/day. This case highlights the importance of ADR as a differential diagnosis and the need of pharmacists with drug safety expertise expert in the patient management.

## Background

Chronic hepatitis B virus (HBV) infection, affecting an estimated 257 million people [1], become one of the most common infectious diseases and a leading cause of liver-related death worldwide. Furthermore, the updated treatment guidelines for chronic HBV management specified that patients had decreased renal function and bone mineral density for a long-term treatment with certain anti-HBV medications [2]. Studies found an association between chronic HBV and renal injury, and increased risk of osteoporosis relative to non-chronic HBV controls [3]. Adefovir dipivoxil (ADV), is an orally bioavailable prodrug of adefovir, used for the management chronic hepatitis B. High-dose ADV therapy of 60–120 mg/day is nephrotoxic and associated with significant rates of renal dysfunction, low-dose ADV of 10 mg/day was reported to be safe [4]. An increasing number of reports stated that use of low-dose ADV for long time caused proximal renal tubular dysfunction, especially in East Asian populations [5–9]. However, at present, there are few cases of renal dysfunction and bone pain caused by adefovir dipivoxil misdiagnosed as cancer or bone tumor, so this article is worthy of clinical reference.

Here, we reported a patient with 27-month history of progressive generalized bone pain combined with spontaneous fractures, who had been suspected as bone tumors or myeloma. Finally, this case was diagnosed by a multidisciplinary clinic as severe hypophosphatemia osteomalacia and renal Fanconi syndrome induced by low-dose ADV.

## Case presentation

### Suspected tumor

In September 2014, the patient developed bone fractures and pain in his bilateral rib cage and ankles and consulted several hospitals to explore what cause the pain. The results of the relevant blood and urine examinations during this period were shown in Table 1. Bone marrow aspiration result showed that the bone marrow was approximately normal. Positron emission tomography combined with a computed tomography (PET/CT) showed increased glucose metabolism in the fifth and seventh ribs and T2 spinous processes on the right side of the body. Based on the PET-CT results, and the probably missed diagnosis of bone tumors due to the location of the bone puncture, a clinical diagnosis of adnexal thoracic tumors was suspected by oncologists. This diagnosis was mainly due to diagnostic method limitation. Based on this diagnosis, posterior adnexal thoracic tumor resection, reconstruction, and internal fixation was performed in the patient’s local hospital in November 2014. Pathological biopsy was performed on the surgical tissues, and the result was as follows: chest-2-appendix hyperplasia, degeneration of cartilage and ligamentum flavum, broken bone trabeculae and bone marrow tissue, trabecular serous fat atrophy, focal hemangiomatous hyperplasia with sinus dilatation. Postoperative pathology showed no tumor cells.

**Table 1:** Blood and urine examination results, 2014

Parameters	Results	Reference Range
Alpha-fetoprotein	2.9	0–20 ng/mL
Carcinoembryonic antigen	2.41	0–6.5 ng/mL
Carbohydrate antigen199	7.6	0–37 u/mL
Prostate specific antigen	0.15	0–4 ng/mL
Carbohydrate antigen 125II	5.6	0–35 u/mL
Carbohydrate antigen − 724	1.18	0–8.2 u/mL
Squamous cell carcinoma-associated antigen	1.94	0–2.5 ng/mL
PTH (parathyroid hormone)	24.4	16–87 pg/mL
Serum light chain λ (bλ-LC)	2.01	0.9–2.1 g/L
Serum light chain К (bκ-LC)	1.65	1.7–3.7 g/L
Hemoglobin (HGB)	138	130-175 g/L
Urue	8.5 ↑	3.2–8.2 mmol/L
Serum creatinine level	154.8 ↑	53–97 umol/L
Phosphorus	0.71↓	0.78–1.65 mmol/L

**Fig. 1:**
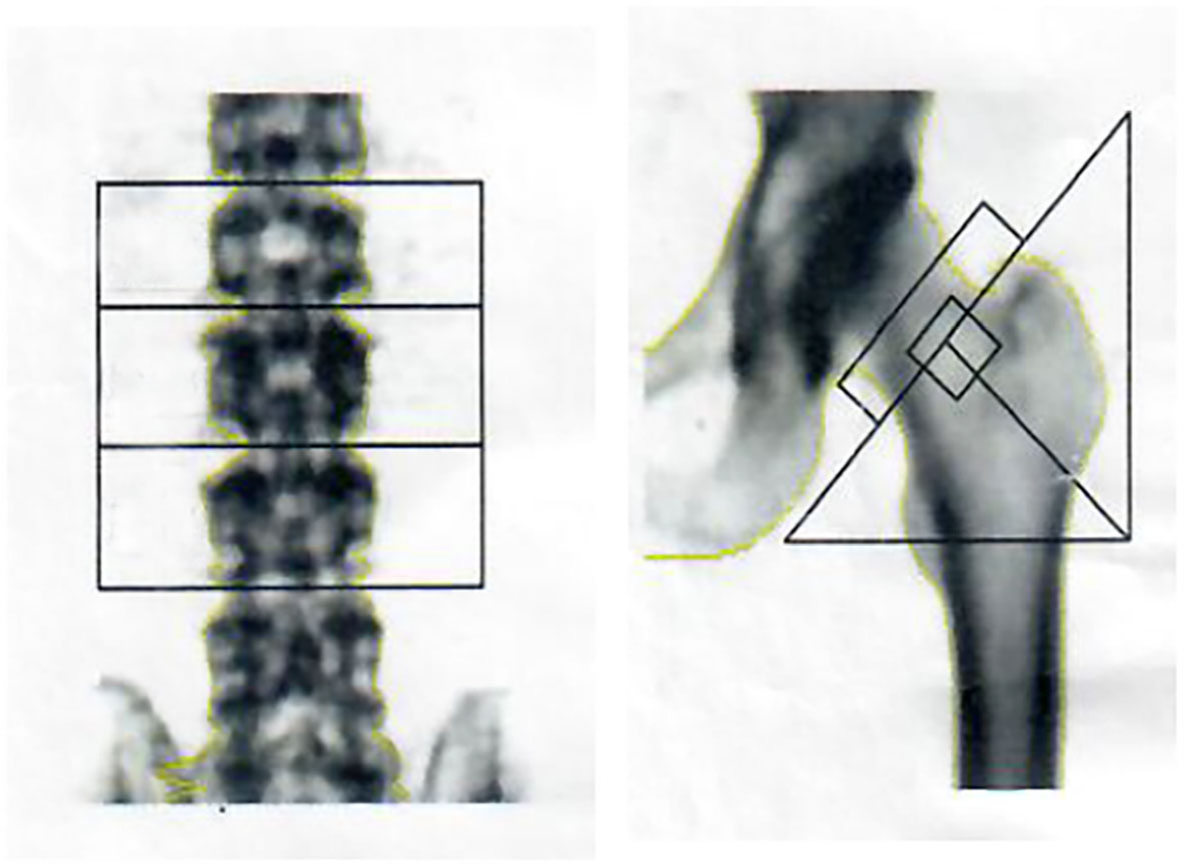
BMD test results in 2016

After surgery, the patient’s bone pain remained and aggravated progressively, and renal function was still poor. Urine and creatinine clearance levels were found to be 9.74 mmol/L (3.2–8.2 mmol/L) and 180.5 μmol/L (53–97 μmol/L), respectively. Because bone marrow aspiration results were negative in 2014, bone marrow biopsy and bone mineral density (BMD) were performed again in 2016 in order to exclude the possibility of bone tumor deterioration. The results of the 2016 bone marrow aspiration showed that the bone marrow was approximately normal: the proliferation of granulocytes was erratic; the proportion and morphology of cells in promyelocytes and the following stages were almost normal; erythrocyte proliferation was active, mainly in the middle and late stages of erythrocyte proliferation, and there was no obvious abnormality in morphology; lymphocytes accounted for 33% of the total blood count, and their morphology was normal; megakaryocytes were also normal. In addition, dual-energy X-ray absorptiometry showed a decreased lumbar spine BMD of 0.817 g/cm^2^ (T-score, − 2.4) and a total BMD of 0.729 g/cm^2^ (T-score, − 2.0) (T T-score < − 2.5, normal > 1.0). The BMD test results are shown in Fig. 1 and Table 2.

**Table 2:** Bone mineral density test results (T score < − 2.5, normal > 1.0) in 2016

Position	Bone mineral density (g/cm2)	T-score of young people	Z-score for normal population of the same age
Lumbar spine 2	0.698	−3.3	−2.5
Lumbar spine 3	0.886	−1.9	− 1.2
Lumbar spine 4	0.847	−2.0	−1.6
Lumbar spine2-Lumbar spine4	0.817	−2.4	−1.7
Neck	0.728	−1.9	−0.9
Triangle of Ward	0.538	−2.3	−1.0
Greater trochanter	0.632	−1.6	−1.1
Femoral shaft	0.808	/	/
Whole	0.729	−2.0	−1.6

Computed tomography and magnetic resonance imaging of the thoracolumbar spine revealed multiple thoracolumbar compression fractures, bilateral multiple rib fractures with calluses, and right iliac wing fractures with calluses. However, serum inorganic phosphorus concentration was low (0.7 mmol/L; 0.78–1.65 mmol/L), alkaline phosphatase (ALP) level was significantly high (161.7; 45–129 IU/mL); urine was 9.23 mmol/L (3.2–8.2 mmol/L), creatinine level was 252 μmol/L (53–97 μmol/L), and eGFR was 22.79 mL/min/1.73 m^2^.

Gene detection was conducted to exclude cancer-related diseases in May 2016. This included detection of Ig gene rearrangement, T-cell receptor gene rearrangement, L256P mutation in the myeloid differentiation factor 88 (MyD88) gene, and qualitative detection of BCL/JH gene rearrangement. However, no genetic cause of bone pain was found, and the pain gradually increased in October 2016. In order to seek further treatment, the patient was referred to the hematology department of the Peking University People’s Hospital, and was recommended by the doctors to visit the multidisciplinary clinic of our hospital. From 2014 to 2016, the patient suffered from bone pain, which he described as ‘physical torture’, and paid about 300,000 RMB in economic expenses, excluding medical insurance reimbursement and transportation costs.

### Multidisciplinary teams and diagnosis of adverse drug reaction

Over the last two decades, referral to the multidisciplinary team (MDT) has become routine procedure in our center, especially for patients with difficult miscellaneous diseases. Clinical pharmacists have begun to participate in the multidisciplinary outpatient clinics in the hematology department since 2016. In January 2017, a patient came to our MDT clinic with 27-month history of progressive generalized bone pain involving severe chest and wall pain, pain in the hips, knees, ankles, and heels. He wanted to explore the reason for his bone pain and discuss further treatment. Physical examination showed tenderness in the middle and back of thoracic vertebrae, both sides of sacroiliac joint, medial side of knee joint and anterior chest wall, with slight spot edema. The neurological symptoms were negative. No stromas were palpable. No specific signs were found in the respiratory, digestive, or circulatory systems. No edema was found in the patient’s legs. No clinical evidence of an infectious, inflammatory, or malignant process were found. Therefore, systemic diseases were excluded. Other related markers, including immunoglobulins tested using immunofixation electrophoresis (IgG/A/M/D), alpha-fetoprotein from 2014 to 2019, were negative (Table 3). Endoscopy did not reveal any mass in the esophagus, stomach or colon. Bone marrow aspiration was normal. Therefore, bone tumors, bone metastasis, or other systemic disease were excluded. The patient had a history of chronic hepatitis B infection, and had received lamivudine at 100 mg/day for 19 years, then changed to 10 mg/day of ADV owing to lamivudine resistance in 2009. After ADV treatment, his liver function was restored.

**Table 3:** Results of various blood and markers tests from 2014 to 2019

Time	AFP^a^	IgG^a^	IgM^a^	IgA^a^	ALT^a^	AST^a^	ALP^a^	LDH-L^a^	TP^a^	ALB^a^	URUE	CR^a^	HGB^a^	Ca^a^	P^a^
	0–20 ng/mL	6.94–16.2 g/L	0.6–2.63 g/L	0.68–3.78 g/L	0–40 u/L	0–34 u/L	45–129 IU/mL	90–250 u/L	60–80 g/L	35–55 g/L	3.2–8.2 mmol/L	53–97 umol/L	130–175 g/L	2.08–2.65 mmol/L	0.78–1.65 mmol/L
2014/10/31	2.9	11.1	1.71	3.29	17.5	20	166↑	121	69.6	44.1	6.6	154.8↑	138	2.16	0.71↓
2015/3/29	2.18				12	15	262↑	148	72.9	42.4	8.5↑	139↑	132	2.26	0.67↓
2016/4/28	1.6	6.87	1.37	3.16											
2016/5/25					21.5	22.4	147.2↑	149.9	64.9	41.9	9.74↑	180.5↑		2.19	0.62↓
2016/6/15					21	23	144↑	152	76	50	10.9↑	175↑		2.4	0.62↓
2016/9/7	1.82				80↑	60↑	173↑	173	72	52		155↑	133		
2016/12/15	2.41	9.17	1.29	3.64	37	33	162↑	142	74.7	47.2		197↑	127	2.1	
2017/1/5	2.43	11.1	1.7	3.74	39	34	149↑	140	83.7↑	50.3	9.23↑	252↑			
2017/1/19					38	38	161.7↑	157	79	52.5	9.0↑	234↑	123	2.5	0.7↓
2017/2/17					49↑	44↑			80	51.5	10.8↑	194↑	123	2.8	0.74↓
2017/5/3					35	38↑		161	78.7	47	9.7↑	192↑		2.33	0.98
2017/12/20	1.81	11.1	1.7	3.74									143		
2018/11/19	2.13				26	30	139↑	170	76.5	50.9	9.5↑	148↑	148	2.48	1.95↑
2019/3/11	1.75				24	19	99	186	73.2	44.9	10.1↑	155↑			

^a^Alpha-fetoprotein (AFP); Immunoglobulin G (Ig G); Immunoglobulin M (Ig M); Immunoglobulin A (Ig A); Alanine transaminase (ALT), Aspartate transaminase (AST); Alkaline phosphatase (ALP); lactate dehydrogenase (LDH); Albumin (ALB); Total protein (TP); Creatinine (CR); Hemoglobin (HGB); Calcium (Ca); Phosphorus (P)

Based on the special situation of the patient, a multidisciplinary consultation discussion was conducted in Peking University First Hospital. During the discussion, doctors differentiated diseases according to their specialty, but could not identify the real cause of the bone pain. Based on the clinical pain manifestations, doctors were suspicious of bone tumors and myeloma, neglecting the possible effects of drugs. Elevated levels of p-enzymes also led doctors to suspect that the patient had a metastatic tumor, while negative monoclonal antibodies in protein electrophoresis excluded the diagnosis of myeloma. As the clinical diagnosis evidence was insufficient, the patient underwent relevant examinations to further confirm whether his bone pain was caused by a bone-related disease. Neither the patient’s test results nor the results of gene screening confirmed bone or cancer-related diseases. On the basis of the medical history and laboratory examinations, the clinical pharmacist suggested a clinical diagnosis of ADV-induced hypophosphatemic osteomalacia due to acquired renal Fanconi syndrome, which was ignored by doctors. The relevant clinical manifestations and diagnostic basis for this patient can be seen in Table 4.

**Table 4:** Relevant clinical manifestations and diagnostic basis of bone tumor, myeloma and adverse drug reactions for this patient

Diagnostic Criteria	Bone tumor	Myeloma	Adverse Drug Reactions	Patient’s condition
Pain, swelling or mass	+	+	+	+
Systemic symptoms				
(anemia, progressive emaciation, insomnia, irritability, ect	+	+	+	+
hepatomegaly	–	+	–	–
renal failure	–	+	+	+
Lymphadenopathy	–	+	–	–
Pathological fracture	+	+	+	+
Bone mineral density examination	–	+	+	+
Protein electrophoresis monoclonal antibody	+	+	–	–
PET/CT				
Skeletal abnormalities in different parts of the body	+	–	+	±
Mainly abnormal lumbar spine	–	+	+	±
Pathological section	–	–	–	–

Note: + means yes, − means no, ± means hard to be sure or negative,? Means that the situation is unknown

Clinical pharmacists paid attention to the situation of drug treatment, and suggested that the bone pain might be a result of drug-induced adverse reactions. Based on the WHO Collaboration Center for International Drug Monitoring (The Uppsala Monitoring Centre) [10] method, the causal relationship of ADR in this case was probable/likely. ADR probability score using Naranjo’s algorithm [11] was 7 points, which is determined as “probable/likely” (5–8 points), as shown in Supplementary Table 1. In addition, preventability assessment using Hallas criteria [12] suggested that it was possibly avoidable. The prescription was not erroneous, but the ADR could have been avoided by an effort exceeding the obligatory demands.

**Fig. 2:**
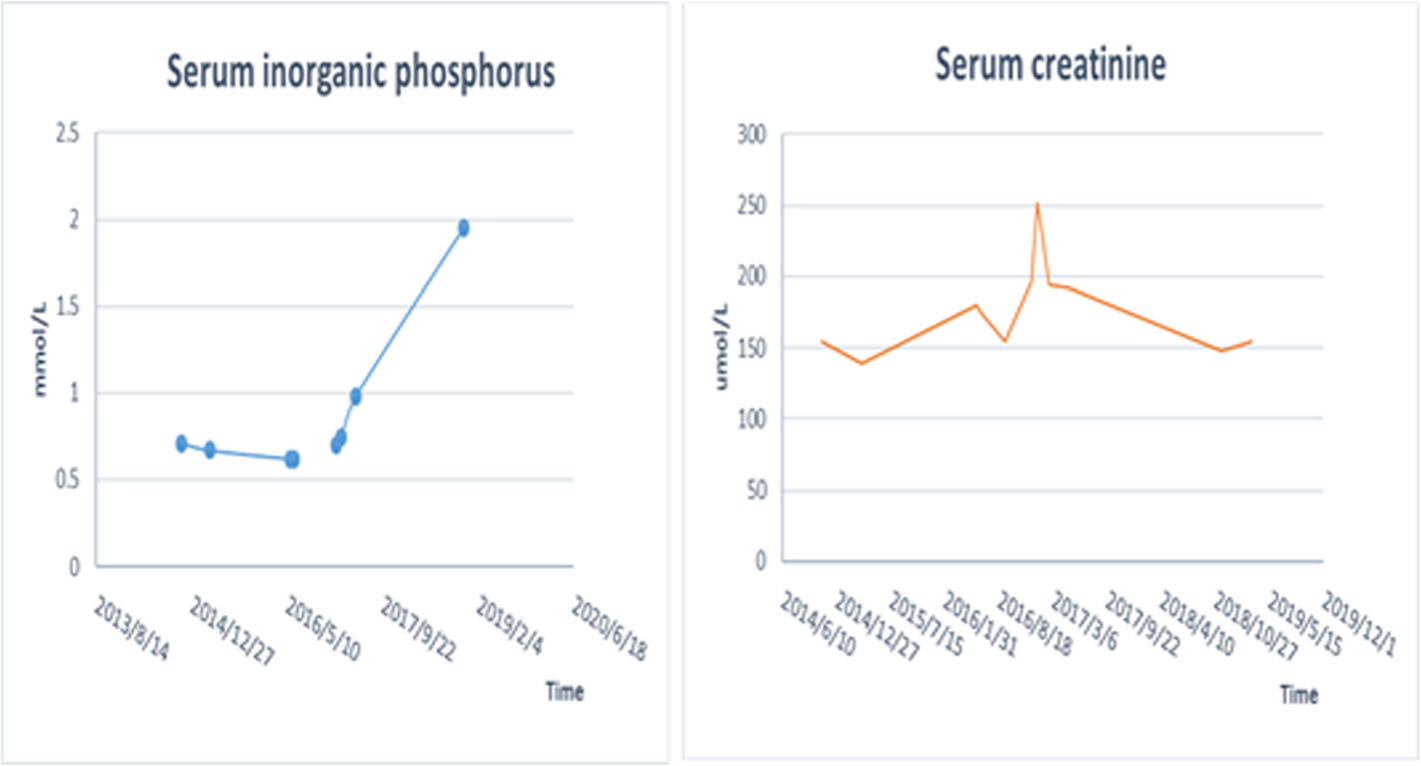
Trend chart of patient’s serum phosphate and creatinine levels from 2013 to 2019

In this case, after the adjustment of anti-HBV drug treatment scheme, the trend of creatinine elevation in the patient was reversed and gradually decreased, but the symptoms of bone pain did not disappear immediately. Bone pain began to relieve after 2 months and disappeared in May 2017. Four months after the discontinuation of ADV and during the 2-year follow-up, the patient’s serum phosphate normalized, and creatinine levels decreased (Fig. 2). Bone pain also disappeared, and his walking ability improved significantly. The dramatic clinical and laboratory improvement observed after ADV discontinuation further supported the diagnosis of ADV-induced hypophosphatemic osteomalacia and renal impairment.

## Discussion and conclusions

Although clinical studies have shown that ADV is generally well tolerated at 10 mg daily with no evidence of proximal renal tubular dysfunction, our case and other studies suggest that actual incidence of ADV-induced hypophosphatemic osteomalacia and renal Fanconi syndrome may well be higher than previously thought.

This paper reports a case of renal and bone damage caused by ADV but misdiagnosed as bone tumors. Progressive bone pain and fracture and abnormal PET-CT are the main reasons for the oncologists suspecting bone tumor. The important of multidisciplinary cooperation and the role of pharmacist in diagnosis and prevention of drug adverse reactions is highlighted in this case. During the whole course of the patient’s medical treatment, the oncologists’ unfamiliarity with adverse reactions to anti-HBV drugs were the main reason for the long-term exposure to the drug and the adverse reaction experienced by the patient.

### Drug-induced Fanconi syndrome

Antiviral drugs are the main cause of Fanconi syndrome. Nucleoside reverse transcriptase inhibitors lead to kidney proximal tubule poisonousness, but it has a higher incidence with the nucleotide reverse transcriptase inhibitors. It might due to the high levels of uptake of these drugs into the proximal tubule cells. Although it is not clear about the mechanism of ADV nephrotoxicity, a likely hypothesis of drug-induced Fanconi syndrome has been proposed [13]. The human organic anion transporter-1 has been demonstrated to mediate the active uptake of ADV from blood into proximal tubular cells [14]. The clinical usage of adefovir and cidofovir has been limited for the nephrotoxicity, and they are known as established causes of Fanconi syndrome [15]. The newer agents with less nephrotoxic, tenofovir and entecavir, and are now the first-line therapy for hepatitis B infection. The recommended dosage of antiviral drugs for different renal impairments, according to the pharmaceutical’s instructions, can be seen in Table 5.

**Table 5:** Recommended doses of antiviral drugs for different renal impairments

Antiviral drugs	Creatinine clearance rate (mL/min)			Hemodialysis
	≥50	30–49	10–29	
Lamivudine	100 mg qd	–	–	–
Adefovir Dipivoxil	10 mg qd	10 mg/48 h (20–49)	10 mg/72 h (10–19)	10 mg/7 days
Entecavir	1 mg qd	0.5 mg qd	0.3 mg qd or 0.5 mg/48 h	0.1 mg qd or 0.5 mg/72 h
Tenofovir Disoproxil Fumarate	300 mg qd	300 mg/48 h	300 mg/(72-96 h)	300 mg/7 days
Telbivudine	600 mg qd	600 mg/48 h	600 mg/72 h	600 mg/96 h

Adefovir is dose-dependent with nephrotoxicity, resulting in renal phosphate consumption and osteomalacia [16]. Osteomalacia is a metabolic bone disease characterized by changes in bone mineralization. Electrolyte abnormalities and osteopenia always lead to muscle weakness, bone pain, fatigue, and pseudofractures found in osteomalacia. Pain start with weight-bearing sites, then spreads to the entire body. Factors identified as predictive of kidney damage and Fanconi syndrome [17] include: age over 40 years, rural environment, renal injury, eGFR < 90 ml/min/1.73 m^2^, hypertension, diabetes, cirrhosis, and ADV treatment exceeding 24 months. Many cases of hypophosphatemic osteomalacia induced by low-dose adefovir (10 mg daily) have been reported [6, 7], especially in Asian countries [18–20].

### Multidisciplinary teams

Disease assessment and management requires complex clinical decision-making, and MDTs participation is encouraged to ensure that a range of professionals with different professional knowledge provide timely and appropriate care [21]. MDT meetings can be defined as regular discussions of patients, including professionals from different disciplines, such as such as surgeons, radiologists, pathologists, nurse specialists, pharmacists, and other health disciplines [22]. In this case report, from 2014 to 2017, the patient suffered from bone pain, sought medical treatment across the country, and experienced pain, which he described as ‘physical torture’, while the economic losses of the patients in the health service accumulated to 300,000 RMB. If MDT meetings were involved in the treatment in 2014, and dosage of ADV was adjusted timely, the patient would have avoided physical pain and personal economic loss for the next 3 years. The patients said that the bone pain gradually disappeared, the quality of life significantly improved, and also saved a lot of medical costs after the medication adjustment.

Fanconi syndrome with osteomalacia can be obtained from patient taking a low dosage (10 mg/day) of ADV. Chronic hepatitis B patients taking ADV (10 mg/day) for long periods of time should pay attention to bone pain and renal function, and regularly monitor indicators of serum ALP, serum phosphorus, serum calcium levels, and bone metabolism markers. Patients with pre-existing renal insufficiency should monitor more frequently. Once ADR is suspected, ADV must be stopped immediately and carried out symptomatic treatment. Similarly, it also requires the differential diagnosis of calcaneal-associated diseases and tumors to prevent misjudgments and affect the diagnosis, treatment, and quality of life of patients.

This case indicates that differential diagnosis of calcaneal-related diseases and tumors is needed to prevent misjudgments and affect the diagnosis, treatment, and quality of life of patients. This case highlights the importance of ADR as a differential diagnosis and the need of pharmacists with drug safety expertise expert in the patient management.

## Supplementary information

**Additional file 1: Table S1** ADR probability scale using Naranjo’s algorithm.

## Data Availability

All data analyzed during this study are included in this published article.

## Abbreviations

ADV: Adefovir dipivoxil
ALP: Alkaline phosphatase
BMD: Bone mineral density
HBV: Hepatitis B virus
hOAT1: Human organic anion transporter-1
PET/CT: Positron emission tomography combined with a computed tomography
MyD88: Myeloid differentiation factor 88
MDT: Multidisciplinary team
