# Supplementary material for: Osteomalacia and renal failure due to Fanconi syndrome caused by long-term low-dose Adefovir Dipivoxil: a case report
Source: BMC Pharmacol Toxicol. 2020 Jun 5;21:43. doi: 10.1186/s40360-020-00421-6 (PMC7275610; doi:10.1186/s40360-020-00421-6)
Supplement: Supplementary file 1 — Additional file 1: Table S1 ADR probability scale using Naranjo’s algorithm. [file 40360_2020_421_MOESM1_ESM.docx]

Supplementary Table 1 ADR probability scale using Naranjo's algorithm.

| Number | Evaluation Criterion | Yes | | No | | Unknow | | | Score |
| --- | --- | --- | --- | --- | --- | --- | --- | --- | --- |
| 1 | Are there previous conclusive reports on this  reaction? | | +1 | | 0 | | 0 | +1 | |
| 2 | Did the adverse event appear after the suspected drug was administered? | | +2 | | -1 | | 0 | +2 | |
| 3 | Did the adverse reaction improve when the drug was discontinued or a specific antagonist was administered? | | +1 | | 0 | | 0 | +1 | |
| 4 | Did the adverse reaction reappear when the drug was re-administered? | | +2 | | -1 | | 0 | 0 | |
| 5 | Are there alternative causes (other than the drug) that could on their own have caused the reaction? | | -1 | | +2 | | 0 | +2 | |
| 6 | Did the reaction reappear when a placebo was given? | | -1 | | +1 | | 0 | 0 | |
| 7 | Was the drug detected in the blood (or other fluids) in concentrations known to be toxic? | | +1 | | 0 | | 0 | 0 | |
| 8 | Was the reaction more severe when the dose was increased, or less severe when the dose was decreased? | | +1 | | 0 | | 0 | 0 | |
| 9 | Did the patient have a similar reaction to the same or similar drugs in any previous exposure? | | +1 | | 0 | | 0 | 0 | |
| 10 | Was the adverse event confirmed by any objective evidence? | | +1 | | 0 | | 0 | +1 | |
| 11 | Total scale | |  | |  | |  | 7 | |
